# Synchrony of Dengue Incidence in Ho Chi Minh City and Bangkok

**DOI:** 10.1371/journal.pntd.0005188

**Published:** 2016-12-29

**Authors:** Cuong Hoang Quoc, Salje Henrik, Rodriguez-Barraquer Isabel, Yoon In-Kyu, Nguyen Van Vinh Chau, Nguyen Thanh Hung, Ha Manh Tuan, Phan Trong Lan, Bridget Willis, Ananda Nisalak, Siripen Kalayanarooj, Derek A. T. Cummings, Cameron P. Simmons

**Affiliations:** 1 Pasteur Institute in Ho Chi Minh City, 167 Pasteur, district 3, Ho Chi Minh City, Vietnam; 2 Oxford University Clinical Research Unit, Hospital for Tropical Diseases, District 5, Ho Chi Minh City, Vietnam; 3 Department of Epidemiology, Johns Hopkins Bloomberg School of Public Health, Baltimore, Maryland, United States of America; 4 Mathematical Modelling of Infectious Diseases Unit, Institute Pasteur, Paris, France; 5 CNRS, URA3012, Paris, France; 6 Center of Bioinformatics, Biostatistics and Integrative Biology, Institut Pasteur, Paris, France; 7 International Vaccine Institute, Seoul, Republic of Korea; 8 Armed Forces Research Institute of Medical Sciences, Bangkok, Thailand; 9 Hospital for Tropical Diseases, District 5, Ho Chi Minh City, Vietnam; 10 Children’s Hospital No. 1, District 10, Ho Chi Minh City, Vietnam; 11 Children’s Hospital No. 2, District 1, Ho Chi Minh City, Vietnam; 12 Queen Sirikit National Institute of Child Health, Bangkok, Thailand; 13 Department of Biology, University of Florida, Gainesville, Florida, United States of America; 14 Department of Microbiology and Immunology, University of Melbourne, at the Peter Doherty Institute, Victoria, Australia; Santa Fe Institute, UNITED STATES

## Abstract

**Background:**

Ho Chi Minh City and Bangkok are highly dengue endemic. The extent to which disease patterns are attributable to local versus regional dynamics remains unclear. To address this gap we compared key transmission parameters across the locations.

**Methods and Principal Findings:**

We used 2003–2009 age-stratified case data to inform catalytic transmission models. Further, we compared the spatial clustering of serotypes within each city. We found that annual case numbers were highly consistent across the two cities (correlation of 0.77, 95% CI: 0.74–0.79) as was the annual force of infection (correlation of 0.57, 95% CI: 0.46–0.68). Serotypes were less similar with serotype-specific correlations ranging from 0.65 for DENV1 to -0.14 for DENV4. Significant spatial clustering of serotypes was observed in HCMC at distances <500m, similar to previous observations from Bangkok.

**Discussions:**

Dengue dynamics are comparable across these two hubs. Low correlation in serotype distribution suggests that similar built environments, vector populations and climate, rather than viral flow drives these observations.

## Author Summary

All four serotypes of dengue have circulated endemically throughout Southeast Asia for decades. However, despite the enormous burden of disease, there remains poor understanding of the similarity in disease patterns across the region. We analyzed data from over 100,000 cases of dengue from two of the largest cities in the region, Bangkok and Ho Chi Minh City between 2001 and 2009. We use basic statistical methods to reconstruct the annual probability of infection in the two cities during this time period using methods that are robust to differences in reporting mechanisms. We find that both the epidemic curves and annual probabilities of infection were highly correlated across the cities, however, serotype-specific correlations were far more variable. Finally, we used geocoded case homes from Ho Chi Minh to demonstrate that cases in the city clustered at spatial scales (<500m) similar to that previously observed in Bangkok. These findings show that dengue dynamics are highly comparable across these two urban hubs; however, the low correlation in serotype distribution suggests that similar built environments and climate, rather than viral flow drives these observations.

## Introduction

Dengue is the most important vector-borne viral disease of humans [1]. It is caused by the four antigenically distinct serotypes (DENV1, DENV2, DENV3, and DENV4). Dengue is a major international public health concern, with an estimated 125 endemic countries [2, 3]. Three quarters of the global population at risk of infection live in the Asia-Pacific region [1, 3]. In particular, Viet Nam has the third highest and Thailand the fifth highest number of annual reported cases in the world [2]. All four serotypes co-circulate in these two countries, with the highest concentration of cases occurring in their largest urban centers, Ho Chi Minh City (HCMC) in Viet Nam and Bangkok in Thailand with more than 100,000 cases reported in each of these cities in 2010 [4, 5].

Despite the substantial number of annual cases, mechanisms that shape the epidemiology of DENV transmission remain unclear. In particular, the importance of local environment in shaping temporal and spatial patterns is not known. Human movement has been implicated in both local and regional dispersion in epidemics in a number of locations; however, population immunity and climatic factors may also have a major role [6]. Comparisons in the dengue experiences across locations can provide insights to disentangle these factors. Understanding how similar dengue epidemics are across settings is also critical for tailoring interventions. Where there is substantial similarity in the transmission patterns of the virus, effective interventions (such as mosquito control efforts, behavioral changes or future vaccines) could have similar impacts across locations.

Previous work using case data has focused on the spread of dengue within urban or rural communities [7–9] or the dispersion of the virus across a country [6]. These studies have demonstrated micro-scale spatial dependence between cases at distances of under one kilometer in both rural Northern Thailand and Bangkok suggesting focal transmission in these settings but also waves of dengue incidence coming out of Bangkok [7, 8, 10]. Phylogenetic studies have found a rate of viral movement between Ho Chi Minh City and surrounding rural communities [11, 12]. On a broader scale, phylogenetic studies have also suggested that Thailand may act as a source of virus for the region, however, these studies suffer from limited availability of sequences and time periods where sequences are available often do not overlap between countries [6, 11]. Ho Chi Minh City and Bangkok are separated by a 90-minute flight (750 km) and are two of the largest and most important regional centers, acting as major trade and airline hubs. They also share similar climates. By comparing the dengue epidemics in the two locations, including serotype distributions and temporal trends in cases, we can explore both the potential inter-dependence of the two settings as well as understand how consistent the dengue experience is in different settings in the same region. Importantly, by using the age distribution of hospitalized cases to estimate the annual probability of infection rather than raw case counts, we can compare disease trends across such locations, even if their surveillance systems are substantially different.

## Materials and Methods

### Ethics statements

All data analyzed in this study were anonymized. This study was approved by the Ethics Committees of the aforementioned three hospitals in Ho Chi Minh City (number 1073/IRB/ Children’s hospital number 1, number 51-2014/39DX/CTU-OUCRU, number 106 IRB/Children’s Hospital number 2, number 106/IRB/Hospital for Tropical Diseases). Analysis of data from Bangkok was approved by ethics committees of the Walter Reed Army Institute of Research, the Queen Sirikit National Institute of Children’s Health and the Johns Hopkins University.

### Study area

Bangkok and Ho Chi Minh City are similar in size, population and climate (Table 1 and S1 Fig). These two urban cities in Southeast Asia share similar characteristics with high population density, high rate of immigrants and typical tropical weather with only two seasons: dry and rainy season.

**Table 1:** Profiles of Ho Chi Minh City and Bangkok

	Ho Chi Minh City	Bangkok
Population, millions	12.9	8.3
Median age, years	29	26
Population density, population per km^2^	3,800	5,300
Mean monthly temperature, C (range)	27 (26–29)	29 (27–31)
Mean monthly precipitation, mm (range)	161(4–312)	137 (6–334)

### Ho Chi Minh City data sources

To understand the proportion of susceptible people that get infected each year in Ho Chi Minh City, we used hospitalized dengue case data from three referral hospitals in the city: Children’s Hospital Number 1, Children’s Hospital Number 2, and the Hospital for Tropical Diseases. Inpatient dengue case burdens reported from these hospitals account for between 95–100% of the dengue surveillance data for HCMC in any given year. For each individual inpatient at one of these three hospitals between January 2001 and December 2009 we used their age and the date of hospital admission for analysis. The infecting serotype was available for 3,609 cases hospitalized at the Hospital for Tropical Diseases over this time period with all four serotypes detected each year [13].

We separately characterized the small-scale spatial dependence of dengue cases in the city using virological data from two clinical studies. The first study (see ClinicalTrials.govID: NCT01550016) was a large prospective outpatient-based study of undifferentiated fever in children/adults at two outpatient clinics in HCMC (Children’s Hospital Number 2, and the Hospital for Tropical Diseases). The 2^nd^ study was also a large prospective study of undifferentiated fever in children across seven outpatient clinics in and around HCMC, including Children’s Hospital Number 1, Children’s Hospital Number 2, and the Hospital for Tropical Diseases. In each study, dengue cases were identified by an RT-PCR diagnostic test on acute plasma samples. We used data from participants enrolled between 2010 and 2013 for this latter study, and from between 2011 and 2013 for the former study. For each individual, we used home address, date of hospital admission and the infecting serotype. Only individuals whose home address was within Ho Chi Minh City were used. All medical and personal data analyzed in this study were anonymized.

#### Adjustment to number of cases observed in Ho Chi Minh City

Of the three study hospitals in Ho Chi Minh City, two were children only hospitals (individuals aged 16 and under) and one hospital cared for individuals of all ages (the Hospital for Tropical Diseases). In order to appropriately pool the data from the three hospitals, we used a scaling factor for adult cases that presented at the Hospital for Tropical Diseases, calculated as follows: We assumed that the probability of individuals sick with dengue presenting at the Hospital for Tropical Diseases was constant for all individuals over 16 years. Further, we assumed that the cumulative incidence of cases by age was approximately linear between the ages of 12 and 20. We calculated the average increase in cumulative incidence by age for each additional year of age for those 12 to 16 using cases from the children’s hospital (β_1_ in the model below).

$$C_{a}=\beta_{0}+\beta_{1}\cdot age$$

where *Ca* is the cumulative incidence at age *a*. We estimated β_0_ and β_1_ using linear regression using individuals between 12 and 16 years only. Using our estimates of β_0_ and β_1_, we calculated the expected cumulative incidence by age at ages 17 to 20. The expected number of cases for each age (*N*_*a*_) between 17 and 20 is then:

$$N_{a}=\frac{{\hat{C}}_{a}-{\hat{C}}_{a-1}}{P_{a}}$$

where *P*_*a*_ is the proportion of individuals in the population of age *a* and was taken from the census data. *N*_*a*_ was calculated for ages between 17 and 20. We then calculated the scaling factor (*SF*) as the average ratio between the expected number of cases and the number of cases observed at the Hospital for Tropical Diseases at those ages.

$$\hat{SF}=0.25*\sum_{i=17}^{20}\frac{N_{a=i}}{N_{a=i}^{HTD}}$$

where $N_{a}^{HTD}$ is the observed number of cases of age *a* at the Hospital for Tropical Diseases. By multiplying the number of observed adult cases at each age by the scaling factor, we obtained an estimate of the total number of cases at that age.

To explore the impact of the scaling factor on our estimates of the force of infection, we conducted a sensitivity analysis where we used a 50% larger scaling factor and separately, a sensitivity analysis where we used a 50% smaller scaling factor.

#### Geocoding addresses in Ho Chi Minh City

We used Google Maps to geocode all addresses from the two cohort studies. Alongside coordinates, this desktop geocoding process provided an indication of uncertainty (either “r*ooftop”*, *“range interpolated”*, *“geometric center” or “approximate”)*. To understand the error for each of these uncertainty levels, we visited 237 of the case homes and collected latitude and longitude coordinates using handheld GPS devices. We then compared the distance between the coordinates from the desktop exercise with those from the handheld GPS devices.

### Bangkok data sources

We used age-specific case data for cases presenting between January 2003 and December 2009 that presented at one of the Bangkok hospitals, made available by the Thailand Ministry of Public Health. For each month, we used the number of DHF dengue cases in each of 14 age groups (under 1 years, 1 years, 2 years, 3 years, 4 years, 5 years, 6 years, 7–9 years, 10–14 years, 15–24 years, 25–34 years, 35–44 years, 45–64 years and over 64 years). Age-specific data were not available before 2003. To characterize the serotype distribution we used previously reported serotype data from Queen Sirikit Children’s hospital, a large hospital in the centre of the city (average of 669 cases with available serotype per year between 2003 and 2009, 6,018 in total) [14]. Descriptions of the spatial distribution of cases in Bangkok have previously been set out [8].

### Statistical analyses

#### Normalized number of annual cases

As case data from Ho Chi Minh City came from three study hospitals while in Bangkok case data came from a surveillance system including all hospitals in the city, to allow appropriate comparison in case trends we normalized the annual number of cases from each city. We normalized the annual number of cases in each location by subtracting the mean annual number of cases for that location and dividing by the standard deviation of the annual number of cases at that location.

We estimated the annual number of cases per serotype per year for each location by multiplying the proportion of cases that were of each serotype by the total number of cases in that location in that year. We used Pearson correlation coefficients to compare both the normalized overall number of cases and the serotype-specific case distributions across the two locations. Confidence intervals for the correlation coefficients were calculated by a bootstrap method. Bootstrap approaches to estimate uncertainty in correlation have been demonstrated to work well with time-series data and incorporate uncertainty in the underlying datasets [15]. Both the serotyped cases and the cases from the surveillance systems from each location were resampled with replacement over 500 iterations with the Pearson correlation coefficient recalculated each time. Ninety-five per cent confidence intervals were generated from the 2.5% and 97.5% quantiles of the resultant distribution.

#### Force of infection

The annual force of infection is the rate at which susceptible individuals are infected in a given year. To generate annual force of infection estimates in both locations we used a method developed by Muench et al. [16] to compare the age distribution of cases between years and has since been applied to dengue [17]. The approach assumes that hospitalised DHF cases tend to be a result of secondary infections. It has previously been estimated that 90% of DHF cases in Bangkok were from secondary infections [18, 19]. We assume that the age distribution of cases represents the age distribution of secondary infections and that everyone is infected at least twice in their lifetime.

Approaches to measure the annual force of infection from the age distribution of cases are well established and have previously been applied to dengue [17, 20]. In particular, Cummings et al., developed methods to calculate the force of infection from hospital incidence data where it is possible to sensibly assume that the majority of cases will represent secondary infections. A major strength of this approach is that it is robust to the proportion of cases observed. This is because it does not use on the number of detected cases but instead relies on the age distribution of cases. Except where the total number of detected cases is very small, the age distribution of cases remains largely robust to the number of cases detected, provided the probability of detecting a case does not vary by age. This facilitates the comparison of forces of infection across locations that may have underlying differences in the probability of detecting cases. Detailed methods are provided in the supplementary materials of Cummings et al [17]. Briefly, the proportion of the population that is age *a* that is completely susceptible to dengue infection in year *y* can be written as:

$$s(a,y)=exp\left[-\sum_{j=0}^{a}4\lambda_{y-j}\right]$$

where *λ*_*y−j*_ is the mean force of infection across the four serotypes in year *y–j*.

The proportion of the population that is age *a* and is infected in year *y* by one of the four serotypes but still susceptible to the other three serotypes (monotypically immune) is then written as:

$$m(a,y)=s(a,y)\cdot \left(exp[\sum_{j=0}^{a}\lambda_{y-j}]-1\right)$$

Finally, those of age *a* that experience a secondary heterotypic infection in year *y* (and therefore more likely to result with severe disease and hospitalisation) is:

z(a,y)=1−s(a,y)−4⋅m(a,y)

If we assume that individuals hospitalized by dengue represent secondary infections, then an individual of age *a* hospitalized in year *y* must have been infected by one serotype in year *y* and by a different serotype sometime in the years between *y-a* and y-1 (assuming you cannot get infected by two serotypes in the same year). The log-likelihood of the cumulative age-specific incidence can therefore be constructed from the susceptible, primary and secondary infections as follows:

$$l(\lambda_{y})=\sum_{i=0}^{A{ge}_{max}}\left\{\left(1-p(a_{i},y))\cdot \log\left[s(a_{i},y)+4\cdot m(a_{i},y)\right]+p(a_{i},y)\cdot log[z(a_{i},y)\right]\right\}$$

where *p*(*a*_*i*_, *y*) is the cumulative proportion of age-specific incidence of those of age *a* in year *y* and age _max_ is the maximum age in the dataset. *p*(*a*_*i*_, *y*) is calculated by dividing the proportion of cases in year *y* that were in individuals of age less than or equal to *a* by the proportion of the population that is of age less than or equal to *a* (as given by census records). In Bangkok, where case data was not available in single year age groups in older individuals, we used the average force of infection for individuals across that age group. We used a quasi-Newton method to identify the maximum likelihood estimate for each *λ*_*y*_ between 2003 and 2009 (years in which we have data from both Bangkok and Ho Chi Minh City) [21]. We assumed a constant force of infection in the years prior to 2003. We used Pearson correlation coefficients to compare the annual force of infection across the two locations. We used country-specific population estimates from both countries to calculate the proportion of the population of each age for each year between 2003 and 2009. To calculate uncertainty we used a bootstrap approach, where all the individuals across the dataset were resampled with replacement. We then recalculated the annual force of infection for each resampling event. Ninety-five per cent confidence intervals were estimated from the 2.5 and 97.5 percentiles of the resultant distribution.

#### Sensitivity analyses

We explored the sensitivity of our force of infection estimates to the number of subsequent years in the dataset. For both Ho Chi Minh City and Bangkok, we deleted between one and five years worth of data from the end of the dataset and recalculated the annual force of infection in each scenario. We also explored the impact where we assumed that 10% or 20% of hospitalized dengue haemorrhagic fever cases were as a result of primary infections with the remainder from secondary infections (the baseline model assumes all cases were as a result of secondary infections). Finally we conducted a sensitivity analysis where we used all case data (i.e., dengue fever cases as well as dengue haemorrhagic fever cases).

#### Basic reproductive number

The basic reproductive number is the mean number of secondary cases generated by one infected individual in a completely susceptible population. This variable therefore provides an indication of the infectiousness of the virus in a particular setting. We estimated the basic reproductive number from the force of infection by the methods from Ferguson et al., [20]. For the calculation of the basic reproductive number we assumed that individuals can only experience up to two infections, and that secondary infection confers full protection against the other circulating serotypes.

#### Small-scale spatial dependence

In addition to understanding the temporal trends in dengue infections in the two cities, we explored whether there were similarities in the spatial clustering of cases. It has previously been demonstrated that there exists small scale clustering of serotypes in Bangkok [8]. In this analysis it was shown that pairs of cases that got infected within the same month had an increased probability of being infected by the same serotype (homotypic infection) when their homes were separated by under one kilometer compared to the probability that any two cases were infected by the same serotype in that month. Here, we used the same statistic (the *τ*–statistic) using geocoded cases that got infected within a month of each other between 2011 and 2013 in Ho Chi Minh City. Addresses with an “approximate” uncertainty level from the geocoding exercise were excluded from the calculation of *τ(d*_*1*_,*d*_*2*_*)* [22]. A sensitivity analysis where only cases with a *‘rooftop’* level was also performed. The estimator can be found in the supplementary materials (S1 Text).

## Results

### Case numbers and serotype distribution

**Fig 1:**
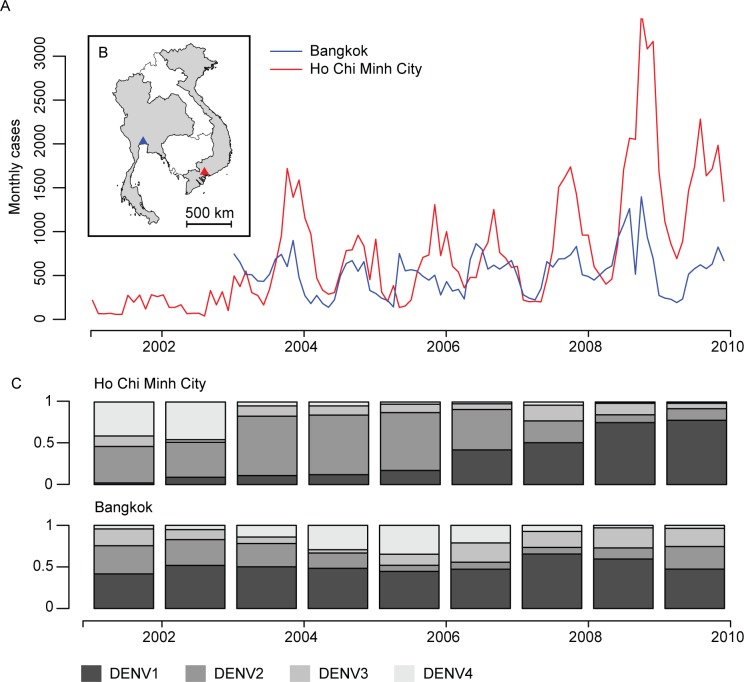
Dengue cases and serotype distribution in Ho Chi Minh City and Bangkok. **A**. Monthly dengue cases distribution, 2001–2010. Red line: dengue cases reported from the surveillance system in Ho Chi Minh City. Blue line: dengue cases reported from Bangkok. **B**. Map showing the location of the two cities. **C**. Dengue serotype distribution in Ho Chi Minh City and Bangkok 2001–2010.

There were 94,864 DHF cases between 2001 and 2009 in Ho Chi Minh City and 46,814 cases in Bangkok between 2003 and 2009. All four serotypes circulated in the two cities during 2001–2009, although DENV1 was the dominant serotype during the entire time series in Bangkok and over the last four years in Ho Chi Minh City (Fig 1). Cases were found throughout the year in both locations, although the majority occurred during the second half of the year (71% of all cases occurred between July and December in Ho Chi Minh City and 61% in Bangkok).

**Fig 2:**
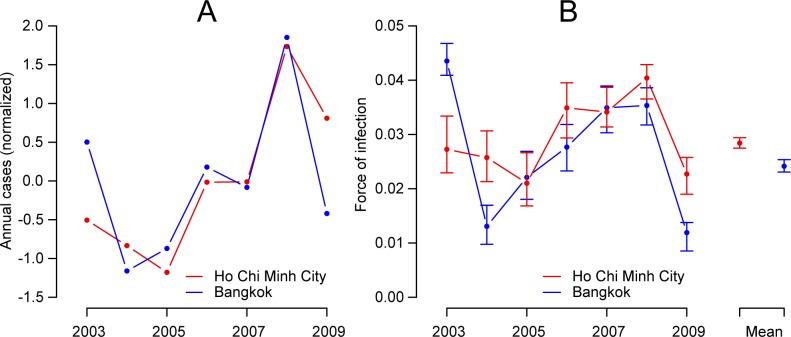
Annual cases distribution and force of infection in two cities, 2003–2009. **A**. Normalized annual dengue cases in Ho Chi Minh City and Bangkok, 2003–2009. Normalized numbers were calculated for each year by removing the overall annual mean number of cases and dividing by the standard deviation. **B.** Annual force of infection in Ho Chi Minh City and in Bangkok, 2003–2009 with 95% bootstrap confidence intervals. On the right is the mean annual force of infection across all years.

**Fig 3:**
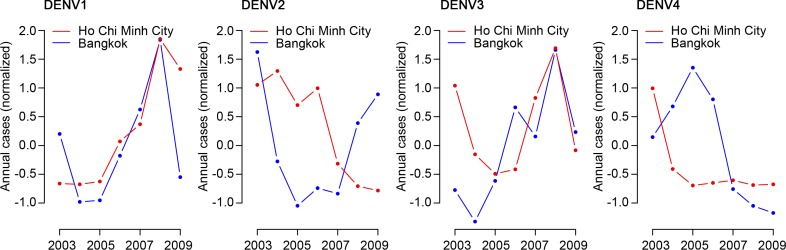
Dengue cases distribution by serotypes between two cities. Normalized annual number of dengue cases in Ho Chi Minh City and Bangkok by serotype, 2003–2009. Initially, raw annual case numbers for each serotype were estimated by multiplying the total number of annual cases in each location (where serotype is generally not available) by the proportion of that serotype from the subset of cases where infecting serotype was identified. Normalized numbers were then calculated for each year by removing the overall annual mean number of cases and dividing by the standard deviation.

The number of annual cases was highly correlated across the two cities (correlation of 0.77, 95% CI: 0.74–0.79) (Fig 2). To explore whether the high correlation in total cases was accompanied by similar serotype distributions across the two locations, we multiplied the estimated proportion of each circulating serotype to the total annual number of cases for each year. We found widely differing levels of correlation by serotype, ranging from high correlation for DENV-1 (the dominant serotype in both locations over the time series) and DENV-3 to no correlation in DENV-2 and negative correlation for DENV-4 (Fig 3). The correlation in annual number of cases was 0.65 (95% CI: 0.54–0.70) for DENV-1, 0.04 (95% CI: -0.19–0.19) for DENV-2, 0.62 (95% CI: 0.35–0.75) for DENV-3 and -0.14 (95% CI: -0.67–0.32) for DENV-4.

### Force of infection and basic reproduction number

**Fig 4:**
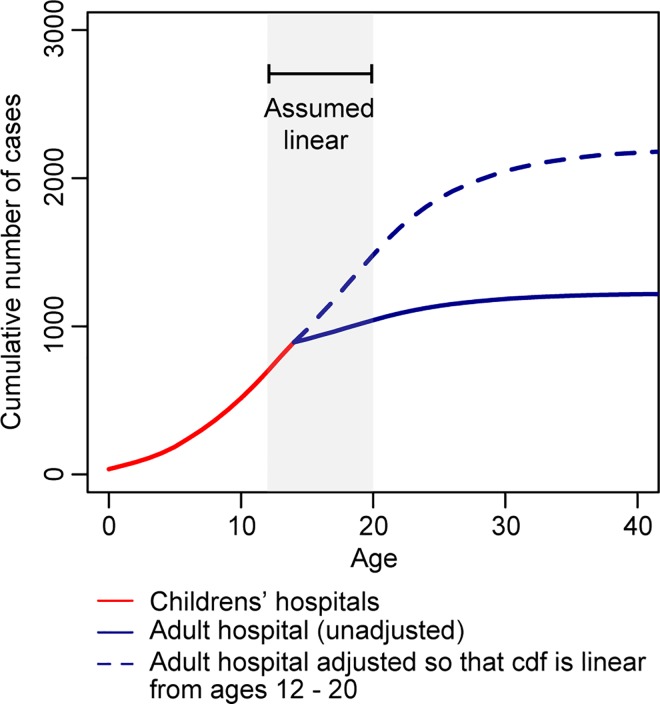
Adjustment to the number of adult cases in Ho Chi Minh City. To appropriately pool case data from two children’s hospitals and a hospital that takes in individuals of all ages, we calculated a scaling factor for adult cases (> 16 years) by assuming the cumulative distribution of cases was linear between those 12 years and those 20 years.

We explored whether the similarity in total number of annual cases across locations was consistent with the annual force of infection over the time series. To calculate the annual force of infection in Ho Chi Minh City, we first had to estimate the number of missing adult cases that would have been detected had they been children (and therefore would have turned up at one of the two children’s hospitals in the study) using the age distribution of all cases in the two hospitals. We estimated that each adult case we observed represented 3.9 total hospitalized cases of that age (see Fig 4 for the adjusted distribution of cases by age).

**Fig 5:**
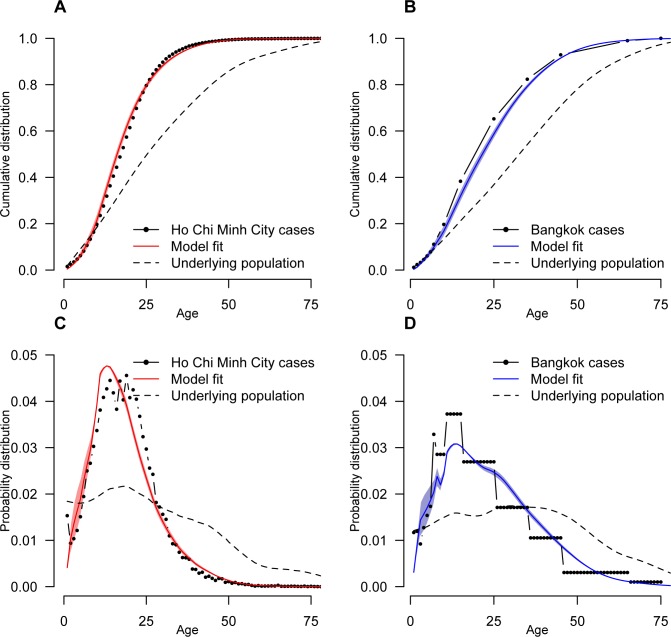
The distribution of ages of cases, adjusting for the age distribution in the country. **A** and **B** represent the cumulative distribution function while **C** and **D** represent the probability density functions. The structure of the underlying population is also plotted in each case. In Bangkok, case data is only available in different sized age groups. In panels **B** and **D**, we give the same value to all ages within the same age group.

Our model was able to broadly capture the observed age distribution of cases in both cities (Fig 5). The mean force of infection between 2003 and 2009 was very similar in the two cities with an estimated 2.9% of the susceptible population infected per serotype each year in Ho Chi Minh City (95% CI: 2.8%-3.0%) compared to 2.4% in Bangkok (95% CI: 2.3% - 2.5%). We estimated that prior to 2003, an average of 3.5% of the susceptible population were infected each year in Ho Chi Minh City (95% CI: 3.4% - 3.5%) compared to 2.2% in Bangkok (95% CI: 2.2% - 2.3%). Further, the pattern in annual force of infections in the two locations tracked each other closely (Fig 2) with a correlation of 0.57 (95% CI 0.46–0.68) between the annual estimates between 2003 and 2009. We used the average force of infection to calculate the basic reproductive number in each city: 3.2 in Bangkok (95% CI: 3.1–3.4) and 3.8 in Ho Chi Minh City (95% CI: 3.6–3.9).

We performed sensitivity analyses where the scaling factor for the number of unobserved cases in Ho Chi Minh City was increased by 50%. This change resulted in a slightly lower mean force of infection between 2003 and 2009 of 2.4% for Ho Chi Minh City (95% CI: 2.3%– 2.5%). The correlation between the annual force of infection estimates between the two cities decreased slightly to 0.51. Decreasing the scaling factor by 50% resulted in an estimated mean force of infection between 2003 and 2009 of 3.7% (95% CI: 3.6%– 3.8%) with a correlation of the annual force of infection estimates between the two cities of 0.53. The annual force of infection estimates from both Ho Chi Minh City and Bangkok were very robust to the use of constrained datasets, where between one and five years worth of data were deleted (S2 Table). In addition, models that assumed that either 10% or 20% of hospitalized dengue hemorrhagic fever cases were from primary infections (the baseline model assumes they are all from secondary infections) did not improve the fit (S4 Fig) as did a models that used all hospitalized case (i.e., including Dengue Fever cases) (S5 Fig).

### Small-scale spatial trend in cases

In addition to exploring the consistency of the temporal patterns of cases, we characterized the small-scale spatial dependence in Ho Chi Minh City using a different dataset of serotyped dengue cases available from cohort studies performed in the same study hospitals during 2010 to 2013. We then compared our findings to a previously published study from Bangkok [8]. In Ho Chi Minh City there were a total of 1444 cases with confirmed serotype and available address information (Table 2). The mean age of participants was 15 (range: 3–60). All four serotypes were present in each year of the study. Overall 36% of cases were DENV-1, 26% were DENV-2, 11% were DENV-3 and 27% were DENV-4. We were able to successfully geocode 1294 of these addresses with a ‘*rooftop*’ (804 addresses), *‘range interpolated’* (245 addresses) or *“geometric center”* (204 addresses) level of accuracy (90% of all addresses). We performed a validation exercise where we visited 237 randomly selected households and recorded locations using handheld GPS recorders. We estimated a median error of 110m for the ‘*rooftop*’ accuracy level, 510m for the *‘range interpolated’* level, 670m for the *‘geometric center’* level and 8.5km for the ‘*approximate*’ level (S2 Table). Addresses with an ‘*approximate*’ accuracy level were excluded from the analysis.

**Table 2:** Overview of cohort studies used in spatial dependence analysis in Ho Chi Minh City

	2010	2011	2012	2013	Overall
Number of participants	168	536	496	244	1444
Study One ^(1)^	0	114	286	225	625
Study Two ^(2)^	168	422	210	19	819
Proportion male	0.45	0.45	0.45	0.37	0.43
Mean age (range)	12 (5–19)	13 (4–47)	17 (3–53)	21 (4–60)	15 (3–60)
No. DENV-1 (%)	88 (52)	212 (40)	146 (29)	71 (29)	517 (36)
No. DENV-2 (%)	54 (32)	172 (32)	104 (21)	47 (19)	377 (26)
No. DENV-3 (%)	20 (12)	74 (14)	42 (8)	21 (9)	157 (11)
No. DENV-4 (%)	6 (4)	78 (15)	204 (41)	105 (43)	393 (27)

^(1)^ Study with participants of all ages with available data between 2011 and 2013.

^(2)^ Pediatric study with data available between 2010 and 2013.

**Fig 6:**
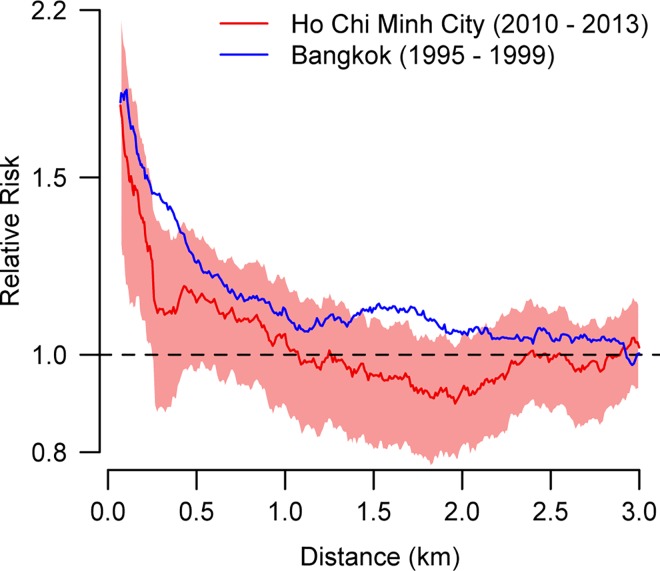
Small-scale spatial dependence for cases occurring in the same month. The relative risk represents the probability of two cases occurring in the same month and separated by a particular distance range (between *d*_*1*_ and *d*_*2*_) being homotypic relative to the probability of any two cases in that month being homotypic. Values greater than 1.0 suggest clustering of serotypes at that distance. The distance range (*d*_*2*_ *–d*_*1*_) was kept constant at 500m. Relative risks are plotted at the midpoint of the spatial range.

We found that in Ho Chi Minh City cases occurring within a month and 200m of each other were 1.6 times more likely to be homotypic than the probability of any two cases being homotypic at that time point (95% CI: 1.2–2.1) (Fig 6). This dropped to 1.0 for cases separated by 750m – 1.25km (95% CI: 0.9–1.2). Significant spatial dependence was observed at distances up to 500m. A consistent pattern was observed when a separate analysis was conducted for each of the study years (S2 Fig). A sensitivity analysis that only used cases with a ‘*rooftop’* uncertainty level also produced very similar results (S3 Fig). The magnitude and extent of spatial dependence observed in Ho Chi Minh City were very similar to previously reported results from Bangkok using hospitalized dengue cases from 1995 to 1999. The Bangkok study found cases occurring within the same month were 1.8 times more likely to be homotypic at distances under 200m (95% CI: 1.5–2.2), although significant spatial dependence was observed at distances up to 1km [8].

## Discussion

This study represents a first attempt to comprehensively compare the spatial and temporal distribution of DENV infection across two of the largest urban centers in Southeast Asia. In these two cities (Ho Chi Minh City in Viet Nam and Bangkok in Thailand), we found remarkably similar patterns of disease and infection over a seven-year period. In particular, we observed a very high correlation in annual case numbers and force of infection between the two cities. This was not, however, accompanied by high correlation in serotype distributions across the two cities. These findings suggest that the similarity in the temporal patterns of dengue was not a result of significant viral flow between the two cities, which we would expect would synchronize serotype distributions. Instead, highly comparable local environments (e.g., high human and vector population density) and supportive local climate (e.g. precipitation and temperature) and possibly regionally climatic factors may drive these similarities. Trends in climatic factors have previously been shown to be correlated with patterns of dengue disease both within Thailand and in the wider Southeast Asian region [23, 24].

The transmission parameters estimated here were consistent with those found using similar approaches from age-stratified case data from different regions of Thailand [17, 25, 26]. It was also consistent with force of infection estimates from seroprevalence studies in Rayong, Thailand (3.8% during [1968–1980], 1.9% during [1993–2010]) in Brazil [27] and cohort studies in Iquitos, Peru (0–33% during [1999–2010]), Ratchaburi, Thailand (6.0% during [2010–2012]) and Viet Nam (3.3% between [2010–2012]) [28–31]. Our findings suggest that in the event that a completely protective vaccine is introduced into Ho Chi Minh City or Bangkok around 70% of the population would need to be effectively immunized in order to prevent pathogen spread.

In addition to consistent temporal patterns in transmission parameters, we also found similar small-scale spatial dependence across the two locations. Serotypes were found to significantly cluster at distances of under 500m in Ho Chi Minh City, similar to that previously estimated in Bangkok using data from a decade earlier. Overall, we observed slightly increased level of spatial dependence in Bangkok, suggesting there may have been small differences in human mobility across these two time periods or across the two locations. This supports a role for focal transmission, even in highly mobile populations, and that while individuals are likely to move substantially on a daily basis, significant levels of infection were happening in and around the home. These findings were compatible with DENV1 local transmission characteristic from a previous study in Viet Nam showing the slow dispersal of the virus in Ho Chi Minh City [32]. Our findings that observing a case was predictive of observing a second homotypic case within the same month at distances of several hundred meters suggests that reactively employed spatially targeted interventions (such as insecticides, which can realistically only be implemented at several tens of meters) may have a limited role irrespective how effective they are.

While our model was able to broadly capture the age-patterns of dengue hemorrhagic fever in both locations, the model fits were not perfect. Allowing some dengue hemorrhagic fever cases to be caused by primary infections or including all cases in the analyses (and not just ones that resulted in hemorrhagic fever) did not improve the fit. This suggests that other factors such age-specific heterogeneity in risk or age-specific heterogeneity in health-seeking patterns may have occurred.

Characterizing uncertainty in the modelling framework we have used is not trivial. By using a bootstrapping approach, we have captured uncertainty in the estimates from the data under our framework. As our analysis makes use of very large datasets (close to 150,000 cases), it is not surprising that uncertainty calculated this way is limited. However, this approach does not capture model (structural) uncertainty. As we are modeling the cumulative incidence proportion, a profile likelihood approach would take account of the likelihood support for the model, however, it would not take account of the size of the dataset being used (i.e., the estimate of uncertainty would be the same if 100 data points were used versus 100,000 data points). Such an approach produces extremely wide confidence intervals (S6 Fig). Overall, neither the bootstrapped nor the profile likelihood confidence intervals provide a truly adequate representation of uncertainty in the parameter estimates and both should be interpreted with caution. Future efforts to estimate the transmission parameters by modeling the incidence rather than the cumulative incidence proportion would allow better quantification of uncertainty.

This study has some further limitations. We assumed that hospital-attended dengue cases represented secondary infections whereas primary infections still occasionally become hospitalized. Primary cases, however, are likely to represent a small proportion of cases in our datasets. A previous hospital-based study in Thailand showed that between 87% and 99% of inpatient dengue cases between 1973 and 1999 had secondary antibody responses [18]. We also only used the more severe DHF cases in both locations in our analyses. Sensitivity analyses that incorporated some primary infections did not improve model fit. In analyzing the Thai and Vietnamese surveillance data we also assumed a similar level of specificity in the classification of dengue cases, i.e. that a similar proportion of the surveillance data represented true dengue cases. We were able to estimate the overall difference in the probability of detecting an adult case versus a child case in Ho Chi Minh City in our datasets. Nevertheless, within these broad age categories (adult and child) and across all ages in Bangkok, we had to assume that the age distribution of detected cases was representative of the age distribution of all secondary infections in the community. Differences in healthcare utilization by age could bias our results and result in higher or lower force of infection estimates. We also used a two-serotype model when estimating the basic reproductive number. The role of tertiary and quaternary infections in transmission remains unclear. Allowing for these infections would reduce our estimates of the basic reproductive number. In an analysis using the same approach as employed here, a study in Rayong, Thailand, found that incorporating tertiary and quaternary infections reduced the estimate of the basic reproductive number from 3.2 to 1.9 [26]. A similar decline would occur here. We did not incorporate temporary cross-protection between serotypes [14]. Allowing for such temporary immunity across other serotypes would increase our force of infection estimates [33]. For the spatial analysis, we had to rely on imperfect desktop geocoding for the majority of cases. Decreased geocoding precision would bias our estimates towards the null suggesting that the true level of spatial dependence may be greater than estimated here.

It is essential that we further determine the factors that drive our observations of high similarity in the dengue epidemic in these two settings. The absence of synchrony in the prevalence of particular virus serotypes, yet reasonably similar overall epidemiological profiles, suggests other influences are important. Thus a more comprehensive approach may be needed, taking into consideration the host, the vector, the virus and other related factors such as micro-climatic conditions, socio-demographic variables, and hygiene to explain more precisely how dengue immunity operates at a small, as well as large scale.

## Supporting Information

S1 Fig Monthly temperature and rainfall for the two cities. A. Comparison of mean temperature between two cities. B. Comparison of monthly rainfall between two cities
(TIF)

S2 Fig Tau clustering statistic by individual year (2010–2013). (TIF)

S3 Fig Tau clustering statistic results when only cases geocoded with ‘ROOFTOP’ accuracy (the most accurate) are used versus when ‘RANGE INTERPOLATED’ and ‘GEOMETRIC CENTER’ are also used. (TIF)

S4 Fig Sensitivity analyses assuming that 0% (baseline scenario), 10% or 20% of hospitalized cases were as a result of primary infections with the remainder as a result of secondary infections. The panels set out the probability density function of the ages of cases under the different models.
(TIF)

S5 Fig Sensitivity analysis using all cases, including both dengue fever and dengue hemorrhagic fever cases. The baseline model uses only dengue hemorrhagic fever cases. The panels set out the probability density function of the ages of cases under the different models.
(TIF)

S6 Fig Uncertainty in the force of infection estimates using profile likelihoods. (TIF)

S1 Table Distance comparison between geocoded address and real GPS (DOCX)

S2 Table Sensitivity exploring impact of removing different years from the dataset (DOCX)

S1 Text Estimator for Tau statistic (DOCX)
