# Correction: Synchrony of Dengue Incidence in Ho Chi Minh City and Bangkok

**DOI:** 10.1371/journal.pntd.0005442

**Published:** 2017-03-17

**Authors:** The PLOS Neglected Tropical Diseases Staff

The second, third and fourth authors have their first and last names in the incorrect order. The correct names, respectively, are: Henrik Salje, Isabel Rodriguez-Barraquer, and In-Kyu Yoon. Also, the ninth author's name is spelled incorrectly. The correct name is: Bridget Wills. The correct citation is: Hoang Quoc C, Salje H, Rodriguez-Barraquer I, Yoon IK, Chau NVV, Hung NT, et al. (2016) Synchrony of Dengue Incidence in Ho Chi Minh City and Bangkok. PLoS Negl Trop Dis 10(12): e0005188. doi:10.1371/journal.pntd.0005188
