# Supplementary figures and images for: Synchrony of Dengue Incidence in Ho Chi Minh City and Bangkok
Source: PLoS Negl Trop Dis. 2016 Dec 29;10(12):e0005188. doi: 10.1371/journal.pntd.0005188 (PMC5199033; doi:10.1371/journal.pntd.0005188)

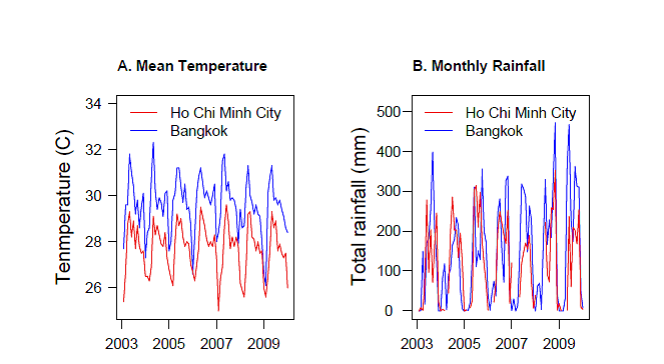

Supplement: S1 Fig — A. Comparison of mean temperature between two cities. B. Comparison of monthly rainfall between two cities (TIF) [file pntd.0005188.s001.tif]

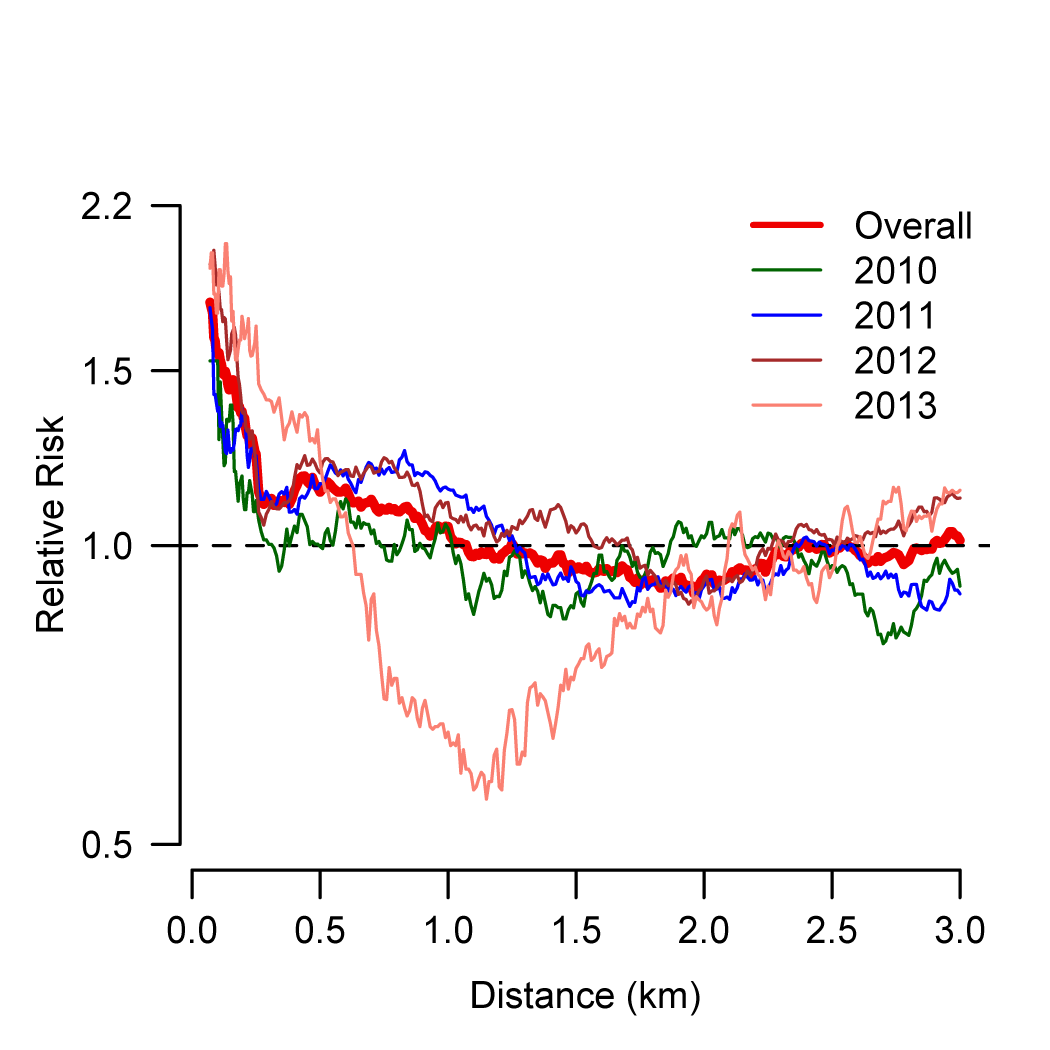

Supplement: S2 Fig — (TIF) [file pntd.0005188.s002.tif]

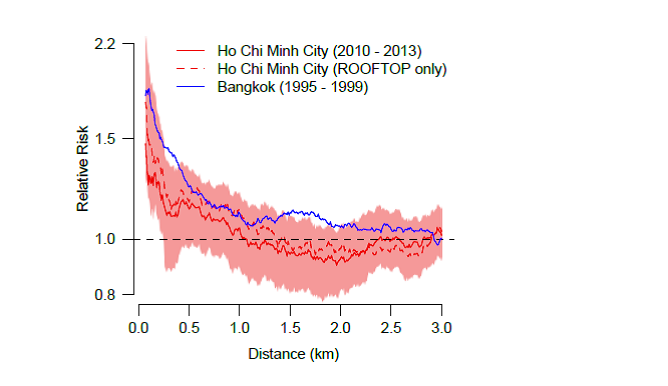

Supplement: S3 Fig — (TIF) [file pntd.0005188.s003.tif]

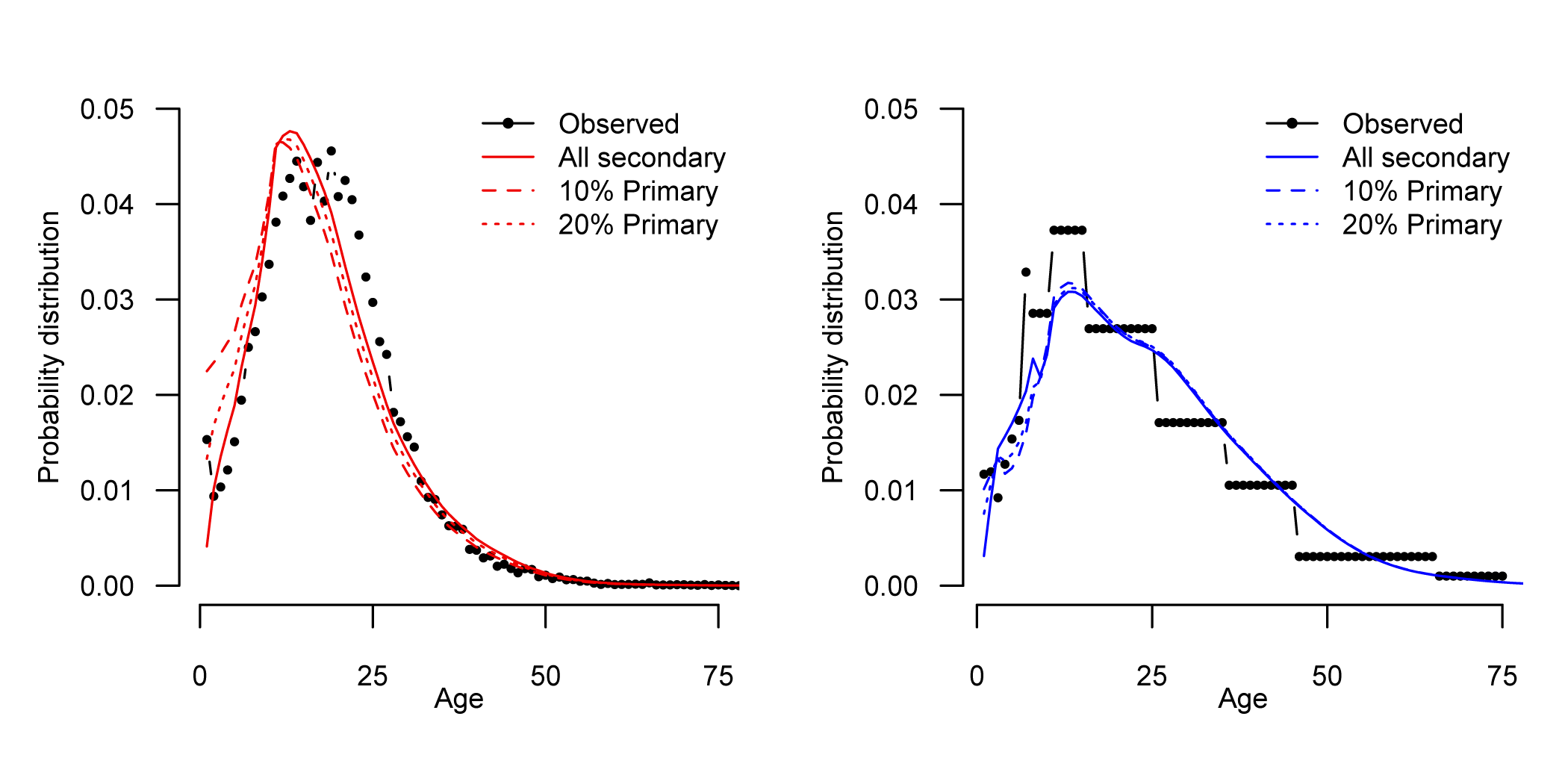

Supplement: S4 Fig — The panels set out the probability density function of the ages of cases under the different models. (TIF) [file pntd.0005188.s004.tif]

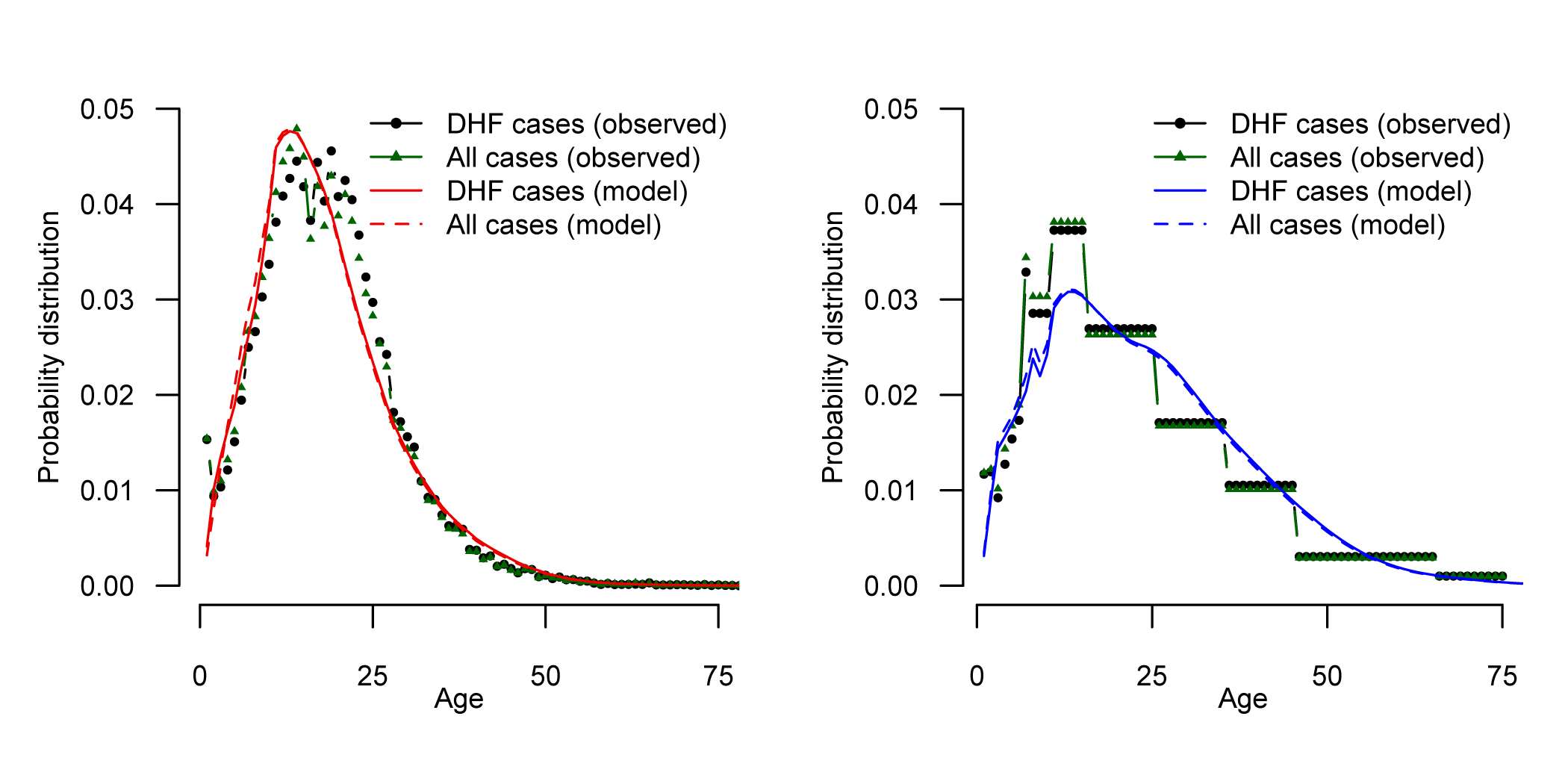

Supplement: S5 Fig — The baseline model uses only dengue hemorrhagic fever cases. The panels set out the probability density function of the ages of cases under the different models. (TIF) [file pntd.0005188.s005.tif]

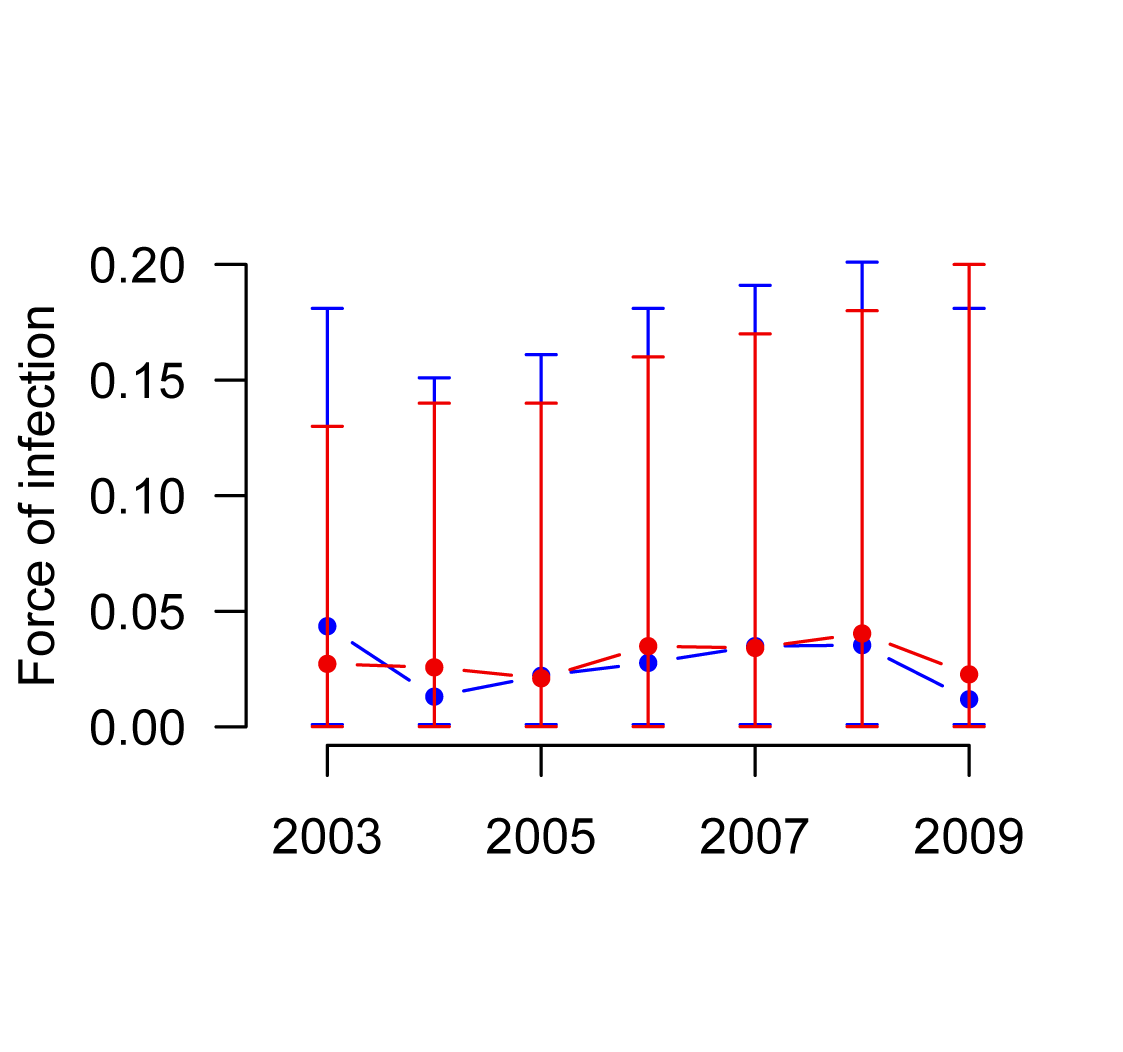

Supplement: S6 Fig — (TIF) [file pntd.0005188.s006.tif]
