# Supplementary material for: Synchrony of Dengue Incidence in Ho Chi Minh City and Bangkok
Source: PLoS Negl Trop Dis. 2016 Dec 29;10(12):e0005188. doi: 10.1371/journal.pntd.0005188 (PMC5199033; doi:10.1371/journal.pntd.0005188)
Supplement: S1 Table — (DOCX) [file pntd.0005188.s007.docx]

**S1 Table: Distance comparison between geocoded address and real GPS**

| **Geocoding type** | **Validation group N** | **Median error (km)** | **Overall dataset N (%)** |
| --- | --- | --- | --- |
| Rooftop | 182 (77%) | 0.11 | 846 (59%) |
| Geometric center | 20 (8%) | 0.51 | 205 (14%) |
| Range interpolated | 24 (10%) | 0.67 | 245 (17%) |
| Approximate | 11 (5%) | 8.51 | 147 (10%) |
| **Total** | 237 (100%) | 0.15 | 1443 (100%) |

**S2 Table: Sensitivity exploring impact of removing different years from the dataset**

**(a) Bangkok**

| **Data used** | **2003** | **2004** | **2005** | **2006** | **2007** | **2008** | **2009** |
| --- | --- | --- | --- | --- | --- | --- | --- |
| **All data**  **(2003-2010)** | 0.044 | 0.013 | 0.022 | 0.028 | 0.035 | 0.035 | 0.012 |
| **2003-09** | 0.044 | 0.013 | 0.023 | 0.029 | 0.037 | 0.040 | 0.012 |
| **2003-08** | 0.042 | 0.013 | 0.023 | 0.030 | 0.040 | 0.052 | - |
| **2003-07** | 0.044 | 0.013 | 0.023 | 0.032 | 0.039 | - | - |
| **2003-06** | 0.047 | 0.013 | 0.024 | 0.033 | - | - | - |
| **2003-05** | 0.050 | 0.013 | 0.024 | - | - | - | - |
| **2003-04** | 0.053 | 0.013 | - | - | - | - | - |

**(b) Ho Chi Minh City**

| **Data used** | **2003** | **2004** | **2005** | **2006** | **2007** | **2008** | **2009** |
| --- | --- | --- | --- | --- | --- | --- | --- |
| **All data**  **(2003-2009)** | 0.027 | 0.026 | 0.021 | 0.035 | 0.034 | 0.040 | 0.023 |
| **2003-08** | 0.027 | 0.027 | 0.020 | 0.036 | 0.036 | 0.045 | - |
| **2003-07** | 0.027 | 0.026 | 0.020 | 0.037 | 0.036 | - | - |
| **2003-06** | 0.029 | 0.026 | 0.021 | 0.038 | - | - | - |
| **2003-05** | 0.028 | 0.027 | 0.021 | - | - | - | - |
| **2003-04** | 0.028 | 0.028 | - | - | - | - | - |

**Appendix Text S1**

**Text S1**

**Estimator for Tau statistic**

The tau statistic used here calculates the probability of a pair of cases from the same month being of the same serotype given they live distance *d* apart, relative to the probability that any two individuals sick in that month are sick with the same serotype:

$$\tau\left( d_{1},d_{2} \right)=\frac{\Pr\left( z_{i}=z_{j} | j\in\Omega_{i}\left( d_{1},d_{2} \right) \right)}{Pr(z_{i}=z_{j}|j\in\Omega_{i}(\cdot))}$$

where *Ω_i_(d_1_, d2)* is the set of cases occurring during the same month and within distances *d_1_* and *d_2_* of case *i; Ω_i_(.)* is the set of all cases occurring in the same month, and *z_i_* is the serotype of case *i*.

The estimator we used for the tau statistic was as follows:

$$\hat{\tau}\left( d_{1},d_{2} \right)=\frac{\sum_{i}^{N} \sum_{i\neq j}^{N} \boldsymbol{I}_{\boldsymbol{1}}(z_{i}=z_{j},s_{ij}<d_{2}, s_{ij}>d_{1},t_{ij}<1 month)}{\sum_{i}^{N} \sum_{i\neq j}^{N} \boldsymbol{I}_{\boldsymbol{2}}(z_{i}=z_{j},t_{ij}<1 month)}$$

where ***I_1_*** and ***I_2_*** are two indicator variables, *s_ij_* is the distance between cases *i* and *j*, *t_ij_* is the time between when the *i* and *j* got sick, *z_i_* is the serotype of case *i* and N is the total number of cases.
