# Supplementary material for: Synchrony of Dengue Incidence in Ho Chi Minh City and Bangkok
Source: PLoS Negl Trop Dis. 2016 Dec 29;10(12):e0005188. doi: 10.1371/journal.pntd.0005188 (PMC5199033; doi:10.1371/journal.pntd.0005188)
Supplement: S2 Table — (DOCX) [file pntd.0005188.s008.docx]

**S2 Table: Sensitivity exploring impact of removing different years from the dataset**

**(a) Bangkok**

| **Data used** | **2003** | **2004** | **2005** | **2006** | **2007** | **2008** | **2009** |
| --- | --- | --- | --- | --- | --- | --- | --- |
| **All data**  **(2003-2010)** | 0.044 | 0.013 | 0.022 | 0.028 | 0.035 | 0.035 | 0.012 |
| **2003-09** | 0.044 | 0.013 | 0.023 | 0.029 | 0.037 | 0.040 | 0.012 |
| **2003-08** | 0.042 | 0.013 | 0.023 | 0.030 | 0.040 | 0.052 | - |
| **2003-07** | 0.044 | 0.013 | 0.023 | 0.032 | 0.039 | - | - |
| **2003-06** | 0.047 | 0.013 | 0.024 | 0.033 | - | - | - |
| **2003-05** | 0.050 | 0.013 | 0.024 | - | - | - | - |
| **2003-04** | 0.053 | 0.013 | - | - | - | - | - |

**(b) Ho Chi Minh City**

| **Data used** | **2003** | **2004** | **2005** | **2006** | **2007** | **2008** | **2009** |
| --- | --- | --- | --- | --- | --- | --- | --- |
| **All data**  **(2003-2009)** | 0.027 | 0.026 | 0.021 | 0.035 | 0.034 | 0.040 | 0.023 |
| **2003-08** | 0.027 | 0.027 | 0.020 | 0.036 | 0.036 | 0.045 | - |
| **2003-07** | 0.027 | 0.026 | 0.020 | 0.037 | 0.036 | - | - |
| **2003-06** | 0.029 | 0.026 | 0.021 | 0.038 | - | - | - |
| **2003-05** | 0.028 | 0.027 | 0.021 | - | - | - | - |
| **2003-04** | 0.028 | 0.028 | - | - | - | - | - |
