# Supplementary material for: Synchrony of Dengue Incidence in Ho Chi Minh City and Bangkok
Source: PLoS Negl Trop Dis. 2016 Dec 29;10(12):e0005188. doi: 10.1371/journal.pntd.0005188 (PMC5199033; doi:10.1371/journal.pntd.0005188)
Supplement: S1 Text — (DOCX) [file pntd.0005188.s009.docx]

**Text S1**

**Estimator for Tau statistic**

The tau statistic used here calculates the probability of a pair of cases from the same month being of the same serotype given they live distance *d* apart, relative to the probability that any two individuals sick in that month are sick with the same serotype:

$$\tau\left( d_{1},d_{2} \right)=\frac{\Pr\left( z_{i}=z_{j} | j\in\Omega_{i}\left( d_{1},d_{2} \right) \right)}{Pr(z_{i}=z_{j}|j\in\Omega_{i}(\cdot))}$$

where *Ω_i_(d_1_, d2)* is the set of cases occurring during the same month and within distances *d_1_* and *d_2_* of case *i; Ω_i_(.)* is the set of all cases occurring in the same month, and *z_i_* is the serotype of case *i*.

The estimator we used for the tau statistic was as follows:

$$\hat{\tau}\left( d_{1},d_{2} \right)=\frac{\sum_{i}^{N} \sum_{i\neq j}^{N} \boldsymbol{I}_{\boldsymbol{1}}(z_{i}=z_{j},s_{ij}<d_{2}, s_{ij}>d_{1},t_{ij}<1 month)}{\sum_{i}^{N} \sum_{i\neq j}^{N} \boldsymbol{I}_{\boldsymbol{2}}(z_{i}=z_{j},t_{ij}<1 month)}$$

where ***I_1_*** and ***I_2_*** are two indicator variables, *s_ij_* is the distance between cases *i* and *j*, *t_ij_* is the time between when the *i* and *j* got sick, *z_i_* is the serotype of case *i* and N is the total number of cases.
